# Unraveling the Relationship Between Seed Yield and Yield-Related Traits in a Diversity Panel of *Brassica juncea* Using Multi-Traits Mixed Model

**DOI:** 10.3389/fpls.2021.651936

**Published:** 2021-05-04

**Authors:** Ranjit Saroj, S. L. Soumya, Satbeer Singh, S. Mukesh Sankar, Rajat Chaudhary, Yashpal, Navinder Saini, Sujata Vasudev, Devendra K. Yadava

**Affiliations:** Division of Genetics, ICAR—Indian Agricultural Research Institute, New Delhi, India

**Keywords:** *Brassica juncea*, mixed model, stepwise regression, path analysis, heritability, germplasm

## Abstract

The response to selection in any crop improvement program depends on the degree of variance and heritability. The objective of the current study was to explain variance and heritability components in Indian mustard *Brassica juncea* (L). Czern & Coss to recognize promising genotypes for effective breeding. Two hundred and eighty-nine diverse accessions of Indian mustard belonging to four continents were analyzed for yield and yield-related traits (20 traits) over two seasons (2017–2018 and 2018–2019) using an alpha lattice design. The genetic variance was found to be significant (*P* ≤ 0.01) for the individual and under pooled analysis for all of the evaluated traits, demonstrating the presence of significant genetic variability in the diversity panel, which bids greater opportunities for utilizing these traits in future breeding programs. High heritability combined with high genetic advance as percent of mean and genotypic coefficient of variation was observed for flowering traits, plant height traits, seed size, and seed yield/plant; hence, a better genetic gain is expected upon the selection of these traits over subsequent generations. Both correlation and stepwise regression analysis indicated that the main shoot length, biological yield, total seed yield, plant height up to the first primary branch, seed size, total siliqua count, days to flowering initiation, plant height at maturity, siliquae on the main shoot, main shoot length, and siliqua length were the most significant contributory traits for seed yield/plant. Also, promising genotypes were identified among the diversity panel, which can be utilized as a donor to improve Indian mustard further. These results indicated a greater scope for improving seed yield per plant directly through a selection of genotypes having the parsimonious combination of these nine traits.

## Introduction

*Brassica juncea* (L.) Czern & Coss (AABB) is the second most important edible oilseed crop in India after the soybean. *B. juncea* is a natural allotetraploid of two diploid species *viz*., *Brassica rapa* (AA) and *Brassica nigra* (BB). Rapeseed is a key species from the *Brassica* genus and a high-value crop for oil and biofuel industries. In India, during 2018–2019, rapeseed–mustard was cultivated over an area of 5.96 million hectares with production and productivity of 8.32 million tons and 1,397 Kg/ha, respectively (Directorate of Economics & Statistics, and Dac&Fw., 2019). Globally, India is the second-largest rapeseed–mustard-cultivating country after China and third in production next to China and Canada (Kumari et al., 2019). In addition to its use as edible oil, mustard oil has a spectrum of industrial utilities such as paint and printing ink additives, greases and lubricants, resins and polymers, plastics, cosmetics, and also in the pharmaceutical industries (Gupta, 2016).

For initiating any genetic improvement program, genetic variability is the prime criterion. Genetic parameters aid in recognizing the gene action and components of genetic variance identification and also facilitate the selection of an appropriate breeding technique. The genotypic and phenotypic variances generally influence the heritability and environmental factors (Bisne et al., 2009). Therefore, the information about heritability and predictability of genetic gains and the association between seed yield and yield-related traits in the base germplasm collection is vital for any genetic improvement program.

Yield is a complex trait and is greatly influenced by environmental factors. Hence, the selection of superior genotypes among a large set of genotypes based on their arithmetic mean performance may not be accurate (Piepho et al., 2008). In this context, the best linear unbiased prediction (BLUP) can provide a good predictive accuracy compared with other procedures for estimating the random effects due to genotypes in a mixed model. It gives a good fit for phenotypic effects to the nongenetic effects by the shrinkage effect toward the probable genetic values. Shrinkage effects by this model anticipate the regression to the mean observed in the selected genotypes, and the individuals having extremely high or low performance consequently got adjusted, thereby improving the accuracy of genotypic effects (Molenaar et al., 2018). Hu (2015) proved that BLUP was effective for calculating genetic parameters and predicting genotypic values and concluded that it could be applied in genetic improvement programs for rapeseed–mustard.

The yield of a crop is directly or indirectly influenced by various yield-contributing traits such as seed size, primary and secondary branches per plant, length of siliqua, seeds per siliqua, etc. Hence, plant breeders often focus on the selection of such traits in combination, each of which was assigned to have a certain level of economic weight based on their importance toward seed yield to form a selection index (Smith, 1936; Hazel, 1943). Multivariate analysis methods, such as genetic correlation analysis, stepwise multiple regression analyses, and path analysis, have been utilized in several crops, including mustard, to identify the causal traits having either direct or indirect effect on seed yield (Olivoto et al., 2016). The path coefficient analysis provides accurate information about the relationship of direct and indirect effects of variables by splitting the correlation coefficients. Therefore, the contribution of each character to yield could be assessed for selecting appropriate traits for indirect selection in any breeding technique (Rao et al., 2013). In contrast, studies using mixed models and sequential path analysis to identify the relationship of cause and effect considering genotypic values in *B. juncea* are still very scarce.

In this context, the current study was carried out with the objectives (i) to use restricted maximum likelihood/BLUP-based method to assess variance, genetic parameters, and genotypic performance of mustard genotypes in multiyear trials, (ii) to fit stepwise regression model for identifying highly significant traits to form a path diagram that explains the relationship of cause and effect among seed yield-related traits, and (iii) to group the germplasm lines sharing a common attribute based on Mahalanobis distance. This study unravels the nature of genetic variability in Indian mustard and would be helpful in the selection of superior genotypes for yield and related traits, which further augment the ongoing and future mustard breeding programs.

## Materials and Methods

### Source of Germplasm and Experimental Location

Two hundred and eighty-nine diverse accessions of *B. juncea* germplasm obtained from Punjab Agricultural University, Ludhiana, under Indian Council of Agricultural Research (ICAR)—National Agricultural Science Fund-sponsored project, including varieties, cultivars, introgression lines, derived lines, and exotic and indigenous collections from the diverse origin such as from India, Australia, Europe, Germany, and Canada were evaluated for phenological and morphological traits under timely sown irrigated conditions. The details of the germplasm accessions used are presented in Supplementary Table 1. The experiments were conducted at ICAR—Indian Agricultural Research Institute (ICAR-IARI), New Delhi, India (latitude—28.708°N, longitude—77.108°E, and altitude—219 m) during 2017–2018 and 2018–2019 winter seasons (October to March).

### Experimental Layout and Observation Recording

Trials were laid out in a randomized alpha lattice design with two replications; each plot consisted of four rows of 2-m length in a plot size of 2.4 m^2^. Five representative plants from each treatment were selected from the middle two rows for evaluation of agronomic performances. Recommended agronomic practices were followed in both seasons. Data of five characters *viz*. days to flowering initiation (DFI), days to 50% flowering (DFF), days to 100% flowering (DHF), days to flowering completion (DCF), and days to maturity (DMT) were recorded on a plot basis. Morphological data of 15 characters recorded on five plants each include plant height at flowering (PH_Fl), plant height up to the first primary branch (PH_FPB), plant height at maturity (PH_M), number of primary branches (PB), number of secondary branches (SB), main shoot length (MSL), siliquae on the main shoot (SMS), total siliquae count (TSC), siliqua length (SL), seeds per siliqua (SPS), seed size (SS), seed yield/plant (SY/Plant), total seed yield/plot (TSY/Plot), biological yield/plot (BY/Plot), and harvest index (HI).

### Statistical Analysis of Phenotypic Data

#### Analysis of Variance

For each given trait, plot-level averages of both seasons were taken as the response variable in an iterative mixed linear model fitting procedure by the full model (Eq. 1) in lme4 R-package (Bates and Maechler, 2009). The best-fit model for each agronomic trait was attained by removing all random terms from the model that were not significant at α = 0.05 in a likelihood ratio test (Littell et al., 2006). Three variance components (σ^2^g, σ^2^gy, and σ^2^e) for each of the 20 traits were calculated using the restricted maximum likelihood (Patterson and Thompson, 1971) estimation method. In the current study, the year was fitted as fixed effect, and genotypes, blocks, replications, and genotype relationship with year were fitted as random effects. The phenotypic results z_*ijkl*_ on accession m in replication k of block l and year i was displayed as:

(1) $$z_{\mathrm{ijkl}}=\mu +y_{i}+g_{j}+r_{\mathrm{ik}}+b_{\mathrm{ikl}}+{\left(\mathrm{gy}\right)}_{\mathrm{ij}}+\varepsilon_{\mathrm{ijkl}}$$

where μ is the grand mean; y_*i*_ is the fixed effect of year i; g_*j*_ is the random effect of genotype, j and is ∼NID(0, σ^2^_*g*_); r_*ik*_ is the random effect of replication, k in year i and is ∼NID(0, σ^2^_*r*_); b_*ikl*_ is the random effect of block l nested with replication k in year i and is ∼ NID(0, σ^2^_*b*_); (gy)_*ij*_ is the random effect of the relations between genotype j and year i and is ∼NID(0, σ^2^_*gy*_); and ε_*ijkl*_ is random residual effect and ∼ NID(0, σ^2^_ε_). Diagnostic residual plots were used to check the normality and homogeneity of the response variable. If the residuals from the fitted model did not meet the assumptions, data were subjected to transformation. This final model was utilized to generate the BLUP for each genotype.

#### Estimation of Heritability and Genetic Parameters

The heritability parameter across the year was estimated by analysis of variance using the ratios of Hallauer and Miranda (1988). The genetic advance was estimated for traits using the formula given by Johnson et al. (1959). The genetic advance as percentage of mean was assessed, as defined by Souza et al. (2009). The phenotypic (PCV) and genotypic coefficients of variation (GCV) were calculated, according to Singh and Chaudhary (2004). Pearson’s correlation coefficient (*r*) between BLUP values for each pair of genotypic traits was estimated using the “corrr” package (Version 0.4.3; Ruiz et al., 2019) in R version 3.5.1 (R Core Team, 2018). To identify the most influential agronomic traits with respect to seed yield/plant as a dependent variable, a stepwise regression model was fitted. The independent variables with the highest share in explaining the variations of the dependent variable were recognized using PAST version 3.09 software. The significance level of a term in the regression model was 5%. R-programs—“agricolae” (de Mendiburu and de Mendiburu, 2019), “Hmisc” (Harrell and Harrell, 2019), and “diagram” (Soetaert, 2009) packages were utilized for path analysis. The divergence was estimated based on the predicted mean (BLUP_*s*_) values of 20 characters pooled over the years, and the residual variance–covariance matrix generated using vcov function of lme4 package was subjected to grouping using the *D*^2^ statistic according to Mahalanobis (1936) and extended by Rao (1952). Mahalanobis’s distance matrix thus obtained was further subjected to clustering by Ward2 hierarchical agglomerative clustering method (Murtagh and Legendre, 2014). R statistical software packages such as “biotools” (da Silva and da Silva, 2017), “dendextend” (Galili, 2015), “circlize” (Gu et al., 2014), “plotrix” (Lemon et al., 2015), “qgraph” (Epskamp et al., 2017), and “car” (Fox et al., 2007) were used for divergence studies based on 20 different agro-morphological traits.

## Results

**FIGURE 1:**
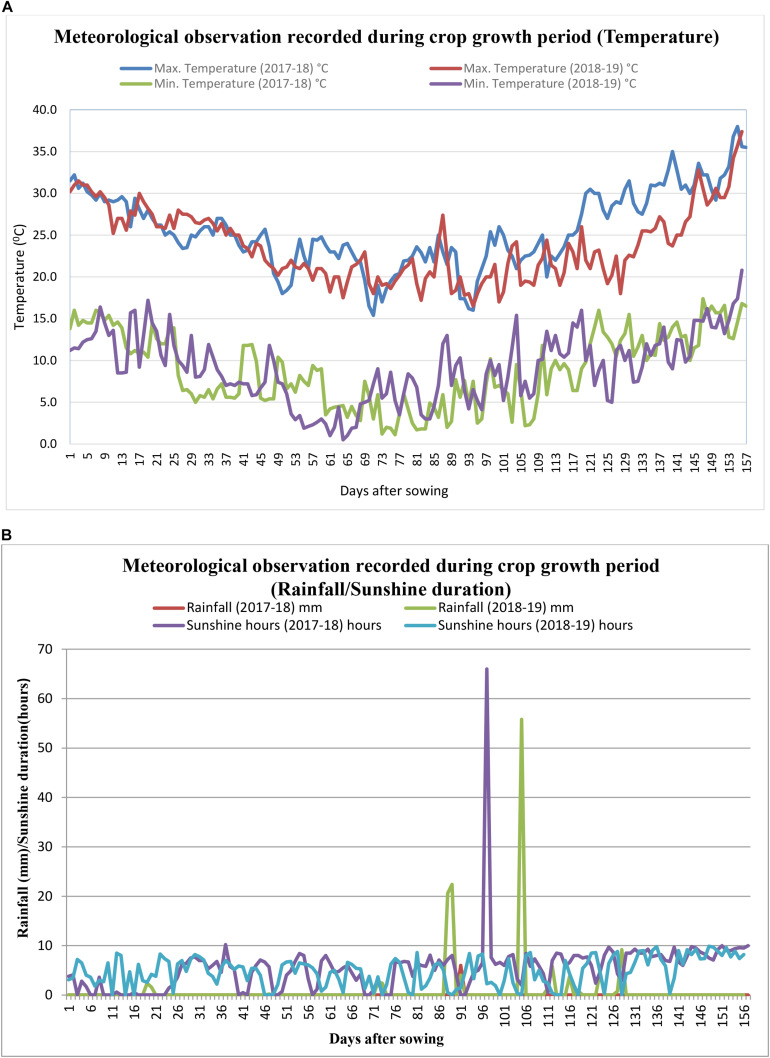
**(A)** Daily maximum and minimum temperatures (°C) recorded during the crop grown period (2017–2018 and 2018–2019). **(B)** Rainfall (mm) and sunshine hours recorded during the crop grown period (2017–2018 and 2018–2019).

Meteorological observations recorded during the season (October–March) are represented in Figures 1A,B. All weather parameters except rainfall were recorded as means over the crop growing period, October–March. Rainfall was recorded as cumulative rainfall received during the period. The average maximum and minimum temperatures were 25.6 and 23.8°C, and 8.9 and 9.2°C during the 2017–2018 and 2018–2019 crop seasons, respectively. During 2017–2018, rainfall and sunshine hours were 6.0 mm and 5.5 h, respectively, whereas, during the 2018–2019 crop season, the average rainfall and sunshine hours were 138.4 mm and 4.8 h, respectively.

### Mean Performance and Variation for Phenotypic Traits

**FIGURE 2:**
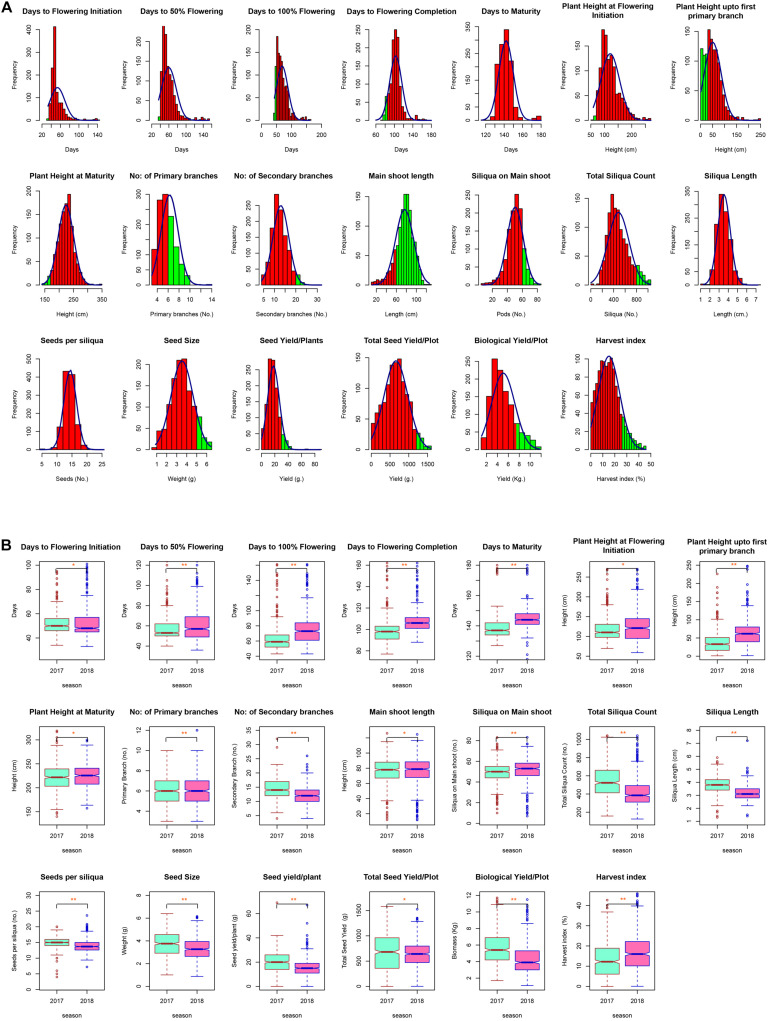
**(A)** Histogram showing distribution of different agro-morphological traits across the diversity panel. Acceptable values for each trait were chosen based on various ideotype concepts given for oilseed *Brassica*, and acceptance region was indicated by green region on the histogram. **(B)** Notched box plots showing difference of 20 traits contributing to grain yield during 2017–2018 and 2018–2019 in New Delhi. Box edges represent upper and lower quartile, with median value shown as a bold line in the middle of the box. Whiskers represent 1.5 times the quartile of the data. Individuals falling outside the range of the whiskers shown as open dots. Boxplot obtained between seasons for each trait was compared using Wilcoxon statistic, and corresponding level of significance was shown by *p*-values in codes (**p ≤ 0.01, *p ≤ 0.05, NS, not significant).

Extensive phenotypic variation was observed for the seed yield and related components under normal sown conditions during both seasons. A large range of variation was observed for most of the traits under study, with the coefficients of variation (CV) ranging from 1.80% for days to maturity to 30.0% for harvest index under normal sown conditions. High CV for some of the yield traits such as TSC, SY/P, TSY/Plot, BY/Plot, and HI was due to longer duration and photoperiod sensitivity of some Canadian accessions—CN-105305, CN-34005, and CN-34008. Most of the traits were approximately normally distributed apart from flowering traits and PH_FPB (Figure 2A). Also, the acceptable level for each trait was indicated by green color on histogram based on the ideotypic concept in mustard given by Bhargava et al. (1984); Thurling (1991), VijayaKumar et al. (1996), Yadav et al. (2017), and DUS guidelines given by Protection of Plant Variety and Farmers Right Authority of India. The boxplots obtained between seasons for each trait were compared using Wilcoxon statistic, and the corresponding level of significance was shown by *p*-values in figures. The analysis indicated a significant mean difference between seasons for each trait. The mean values of all traits except siliquae on the main shoot and harvest index were slightly higher in the season 2017–2018 than 2018–2019 (Figure 2B). The mean performance of SY/P during 2017–2018 was 20.38 ± 0.4 g, whereas, during 2018–2019, it was 15.38 ± 0.3 g. Based on the mean performance, IC-597867 yielded the highest seed yield/plant of 78.5 g per plant, and CN-34005 has no yield during 2017–2018. Similarly, IC-597867 remains the highest yielder of 59.5 g per plant, whereas CN-105364 yielded the lowest of 1 g/per plant. The best performing genotype across the year was IC-597867, whereas CN-105364 was the least performer. An overview of the agro-morphological traits recorded pooled over 2 years is shown in Table 1.

**TABLE 1:** An overview of the agro-morphological traits recorded pooled over 2 years.

Parameters	Mean	Range				SE(m)	CV	LSD	No. of genotypes above trial mean
		
		Min. value	Genotype	Max. value	Genotype				
Days to flowering initiation	53.5	33.5	DTM−25	139	CN−105364	0.47	5.23	10.67	94
Days to 50% flowering	59.71	38.5	DTM−25	153	CN−105364	0.51	4.85	11.17	107
Days to 100% flowering	68.69	44.0	DRMRIJ 17−46	160.5	CN−105364	0.57	5.41	12.47	120
Days to flowering completion	102.88	84.75	EJ−17	166	CN−105364	0.37	3.69	7.42	133
Days to maturity	142.12	126.5	IC−261627	179	CN−105364	0.23	1.80	4.63	121
Plant height at flowering	121.09	77.9	GR−325	268.9	CN−105379	1.01	9.45	24.99	131
Plant height up to the first primary branch	50.58	7.8	NRCHB−101	225.3	CN−105379	1.03	19.69	24.99	127
Plant height at maturity	223.74	165.56	IC−261627	320.3	CN−105379	0.80	5.25	20.4	151
Number of primary branches	6.27	4	PBR−378	11	AJ−2	0.05	14.53	1.07	138
Number of secondary branches	13	5	CN−105305	20.25	AJ−2	0.11	18.10	1.8	136
Main shoot length	76.53	14	CN−105257	107.06	RNN−505	0.53	11.26	12.61	151
Siliquae on the main shoot	50.46	12.85	CN−105257	74.3	IC−597885	0.31	13.19	7.68	158
Total siliquae count	483.56	173	CN−105305	904.5	AJ−11	5.39	24.12	116.7	135
Siliqua length	3.53	1.4	CN−105257	5.1	NRCDR−2	0.02	7.62	0.41	137
Seeds per siliqua	14.38	8.65	CN−105379	19.7	RE−35−4	0.06	11.19	1.25	152
Seed size	3.53	1.1	CN−113780	6.22	RH−406	0.03	6.52	0.76	143
Seed yield/plant	17.88	1.3	CN−34008	69.67	IC−597867	0.26	27.78	6.23	140
Total seed yield/plot	657.15	34.5	CN−34008	1275	RGN−73	9.36	23.13	220.94	148
Biological yield/plot	5.06	1.8	CN−113780	10.28	CS−54	0.06	25.95	1.28	148

*SE(m) is the standard error of mean, CV is coefficient of variation, and LSD is least square difference.*

Significant effects of the sources of variation are presented in Table 2. The effect of genotypes was significant (*P* ≤ 0.01) for all the studied traits for both years, representing the presence of considerable genetic variability in the germplasm for all the traits. Significant G × E interactions were observed for all the traits during pooled analysis, suggesting different genotypes response to environmental conditions differentially.

**TABLE 2:** Genotypic variance (σ^2^_*g*_), genotypic–year interaction variance (σ^2^_*gy*_), standard error (SE), and heritability in broad-sense (H^2^) estimated over each season and pooled data along with pooled mean values, genetic and phenotypic coefficients of variation (GCV and PCV), genetic advance (GA), and genetic advance as percentage of mean for the 20 traits studied.

Characters	2017–2018			2018–2019			Pooled analysis										
			
	σ^2^_*g*_	SE	H^2^	σ^2^_*g*_	SE	H^2^	σ^2^_*g*_	SE	σ^2^_*gy*_	SE	H^2^	Mean	σ^2^_*p*_	GCV	PCV	GA	GA% mean
DFI	204.11**	8.49	0.98	292.47**	12.17	0.99	181.12**	5.33	66.07**	1.94	0.84	53.5	255.47	25.15	29.87	27.6	51.6
DFF	232.65**	9.68	0.98	338.66**	14.09	0.99	214.02**	6.29	71.29**	2.1	0.85	59.71	294.84	24.5	28.76	30.1	50.4
DHF	275.26**	11.45	0.98	384.12**	15.98	0.98	240.54**	7.07	89.13**	2.62	0.83	68.69	344.92	22.58	27.04	31.9	46.4
DCF	132.71**	5.52	0.93	113.30**	4.71	0.96	97.78**	2.88	25.62**	0.75	0.86	102.88	138.48	9.61	11.44	20.8	20.2
DMT	47.13**	1.96	0.94	40.01**	1.66	0.92	33.72**	0.99	9.77**	0.29	0.84	142.12	50.69	4.09	5.01	12.3	8.6
PH_Fl	809.98**	33.69	0.92	1258.82**	52.36	0.95	680.44**	20.01	354.17**	10.42	0.76	121.09	1174.88	21.54	28.31	54	44.6
PH_FPB	740.72**	30.81	0.93	1170.72**	48.7	0.96	551.59**	16.22	404.01**	11.88	0.71	50.58	1060.49	46.43	64.38	47.5	94
PH_M	611.16**	25.42	0.87	559.88**	23.29	0.93	350.78**	10.32	236.86**	6.97	0.70	223.74	732.96	8.37	12.1	38.8	17.4
PB	0.99**	0.04	0.67	1.76**	0.07	0.84	0.56**	0.02	0.81**	0.02	0.48	6.27	2.31	11.94	24.23	1.5	23.8
SB	4.73**	0.2	0.55	7.08**	0.29	0.80	1.07**	0.03	4.82**	0.14	0.22	13	11.83	7.95	26.45	1.6	12
MSL	190.98**	7.94	0.78	314.70**	13.09	0.94	180.52**	5.31	68.76**	2.02	0.77	76.53	323.57	17.56	23.51	28.7	37.4
SMS	55.39**	2.3	0.68	78.67**	3.27	0.81	41.52**	1.22	25.48**	0.75	0.64	50.46	112.09	12.77	20.98	13.9	27.5
TSC	13826.75**	575.12	0.60	15146.49**	630.01	0.78	6653.75**	195.7	7807.28**	229.63	0.48	483.56	28794.07	16.87	35.09	166.6	34.5
SL	0.32**	0.01	0.89	0.23**	0.01	0.87	0.20**	0.01	0.07**	0	0.79	3.53	0.36	12.67	17.02	1	27.7
SPS	2.52**	0.1	0.81	0.71**	0.03	0.26	0.63**	0.02	0.99**	0.03	0.36	14.38	4.23	5.52	14.3	1.5	10.5
SS	1.19**	0.05	0.96	1.06**	0.04	1	0.78**	0.02	0.34**	0.01	0.81	3.53	1.18	25.03	30.8	1.8	51.4
SY/Plant	62.04**	2.58	0.81	29.99**	1.25	0.74	26.67**	0.78	19.64**	0.58	0.63	17.88	71.26	28.88	47.21	10.9	60.8
TSY/Plot	110225.70**	4584.78	0.88	42614.75**	1772.54	0.83	19844.99**	583.68	56780.60**	1670.02	0.37	657.15	101648.9	21.44	48.52	241.3	36.7
BY/Plot	2.16**	0.09	0.65	1.77**	0.07	0.76	0.70**	0.02	1.26**	0.04	0.40	5.06	3.93	16.54	39.21	1.6	32
HI	47.96**	1.99	0.81	45.42**	1.89	0.79	8.06**	0.24	38.96**	1.15	0.24	15.09	74.8	18.82	57.32	4.3	28.5

**is the level of significance at 1%.

*DFI, days to flowering initiation; DFF, days to 50% flowering; DHF, days to 100% flowering; DCF, days to flowering completion; DMT, days to maturity; PH_Fl, plant height at flowering (PH_Fl), PH_FPB, plant height up to first primary branch; PH_M, plant height at maturity; PB, number of primary branches; SB, number of secondary branches; MSL, main shoot length; SMS, siliquae on the main shoot; TSC, total siliquae count; SL, siliqua length; SPS, seeds per siliqua; SS, seed size; SY/Plant, seed yield/plant; TSY/Plot, total seed yield/plot; BY/Plot, biological yield/plot; HI, harvest index.*

### Estimation of Heritability and Genetic Parameters

The phenotypic component of the variance was divided into genotypic variance (σ^2^_*g*_), G × E variance (σ^2^_*gy*_), and error variance (σ^2^_*e*_). Furthermore, genotypic and G × E variances were compared with total phenotypic variance to identify the magnitude of genotypic contribution for *Brassica* improvement. In the present study, most of the traits were highly heritable (>0.60) as per the scale of Robinson (1966) in combined environments except SB and HI, which showed low heritability (>0.30). Similarly, PB, TSC, SPS, TSY/Plot, and BY/Plot showed moderate heritability (Table 2). The estimates of broad-sense heritability in pooled data ranged from 0.22 (SB) to 0.86 (DCF). Traits such as flowering, plant height-related, siliquae on the main shoot, siliqua length, seed size, and seed yield/plant were found to be more heritable. For seed yield/plant, the heritability was high in first year (2017–2018) compared with second year (2018–2019), whereas combined analysis resulted in the lowest value for heritability, indicating the significant partitioning of G × E variance from genetic variance for these traits obtained in the individual environment.

The PCV and GCV, genetic advance, and GA as % mean were calculated along with heritability for all the traits (Table 2). The highest GCV and PCV were observed for plant height up to the first primary branch (46.43 and 64.38%, respectively), and the lowest GCV and PCV were recorded for days to maturity (4.09 and 5.01%, respectively). Results suggested a narrow difference between GCV and PCV for highly heritable phenological traits such as DFI, DFF, DHF, DCF, DMT, PH_Fl, PH_M, MSL, etc., which can be improved directly by selecting genotypes having a higher trait value. However, the difference is more prominent for traits with low heritability, such as PB, SB, TS, BY, SY/P, and TSY/Plot, indicating the significant influence of environment and G × E interactions. In all these traits, PCV was considerably higher than GCV. The genetic advance as a percentage of the mean ranged from 8.6% in DMT to 94.0% in PH_FPB. The results showed that selecting the top 5% of the genotypes could result in genetic improvement of 51.6% for DFI, 50.4% for DFF, and so on (Table 2). The current study found that among the parameters under study, high heritability (≥60%), genetic advance as percentage mean (>20%), PCV (>20%), and GCV (>20%) were observed in characters such as days to flowering initiation, days to 50% flowering, days to 100% flowering, plant height at flowering, plant height up to the first primary branch, seed size, and seed yield/plant. High PCV, GCV, and genetic advance values with low and moderate heritability were observed for total seed yield/plot, biological yield/plot, and harvest index.

### Association Among Traits and Their Contribution Toward Seed Yield per Plant

**FIGURE 3:**
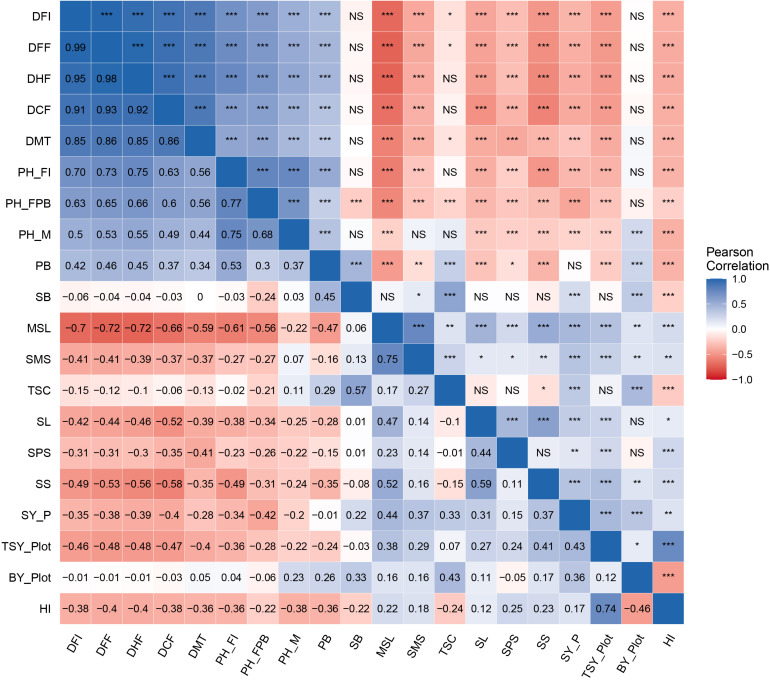
Heat map of genotypic correlation matrix for yield and yield-contributing traits in *Brassica juncea* genotypes. Scale on the side of the figure indicates magnitude and direction of phenotypic correlations. Shades of blue from lighter to darker indicates strength of positive correlation between pairs of traits. Shades of red from lighter to darker indicates strength of negative correlation between pairs of traits. Lighter to white color indicates very weak or no correlation between pair of traits. Pearson’s correlation coefficients (lower diagonal) and significance (upper diagonal) among different traits measured in this study for genotypic BLUP values. Significance codes: ***p ≤ 0.001, **p ≤ 0.01, *p ≤ 0.05, NS, not significant.

The utility of independent traits in the selection can be expected by their significant association with seed yield (dependent trait). In the present study, genotypic correlations between 20 character pairs were studied in all possible combinations (Figure 3). The prime economic trait, seed yield per plant, was positively and significantly correlated (*p* ≤ 0.01) with total seed yield per plot (*r* = 0.43), seed size (*r* = 0.37), main shoot length (*r* = 0.44), siliquae on main shoot (*r* = 0.37), siliqua length (*r* = 0.31), seeds per siliqua (*r* = 0.15), total siliquae count (*r* = 0.33), number of secondary branches (*r* = 0.22), biological yield per plot (*r* = 0.36), and harvest index (*r* = 0.17). Seed yield per plant exhibited a significant negative correlation with flowering characters such as days to 50% flowering (*r* = −0.38), days to 100% flowering (*r* = −0.39), days to flowering completion (*r* = −0.40), days to flowering initiation (*r* = −0.35), days to maturity (*r* = −0.28), and plant height characters such as plant height at flowering initiation (*r* = −0.34), plant height up to the first primary branch (*r* = −0.42), and plant height at maturity (*r* = −0.20), whereas number of primary branches showed a nonsignificant relationship.

A stepwise regression model with seed yield/plant as a dependent variable and other traits as the independent variable identified BY/Plot, TSY/Plot, PH_FPB, SS, TSC, DFI, PH_M, SMS, and SL as highly significant traits. However, keeping seed yield per plant as a dependent variable, these traits with compounded effect explained approximately 46% of total variance with an *R*^2^ value of 0.46 (Table 3).

**TABLE 3:** Summary of stepwise regression analysis considering seed yield per plant as a dependent variable to determine the significance and relative contribution of other traits under normal sown condition.

Variable	Method	AIC	RSS	Sum Sq	*R*^2^	Adj *R*^2^
MSL	+	–59.96	231.62	56.38	0.20	0.19
BY/Plot	+	–89.87	207.41	24.21	0.28	0.27
TSY/Plot	+	–118.50	186.55	20.86	0.35	0.35
PH_FPB	+	–134.41	175.34	11.21	0.39	0.38
SS	+	–146.39	165.91	2.54	0.42	0.41
TSC	+	–151.25	162.02	3.89	0.44	0.42
DFI	+	–153.34	159.74	2.28	0.45	0.43
PH_M	+	–155.37	157.53	2.21	0.45	0.44
SMS	+	–156.83	155.66	1.88	0.46	0.44
MSL	−	–157.79	156.22	0.56	0.46	0.44
SL	+	–158.64	154.68	1.53	0.46	0.44

*“+” means forward selection, and “–” means backward selection.*

*AIC, Akaike information criterion; RSS, residual sum of squares; *R*^2^, coefficient of determination; MSL, main shoot length; BY/Plot, biological yield/plot; TSY/Plot, total seed yield/plot; PH_FPB, plant height up to first primary branch, SS, seed size; TSC, total siliqua count; DFI, days to flowering initiation; PH_M, plant height at maturity; SMS, siliquae on the main shoot; SL, siliqua length.*

The path analysis (Table 4) showed that the total seed yield/plot had the highest positive direct effect (ρ_*X1*_ = 0.242) followed by siliquae on the main shoot (ρ_*X4*_ = 0.225) and seed size (ρ_*X5*_ = 0.215). However, this trait had an important negative direct effect through plant height up to the first primary branch (ρ_*X2*_ = −0.185) and plant height at maturity (ρ_*X9*_ = −0.150). Siliqua length and biological yield/plot showed a positive indirect effect on yield (ρ = 0.216 and ρ = 0.203, respectively). However, days to flowering initiation (ρ = −0.350) and plant height up to the first primary branch (ρ = −0.235) contributed with negative indirect effects on seed yield. The present study also entrusted to identify promising genotypes for significantly associated traits identified by regression and path analysis (Table 5). Some of the genotypes showed superior performance for more than one trait, *viz*. IC-597867 is found to be the potential donor for traits such as basal branching, total siliquae count, and seed yield per plant. Similarly, PBR-210 and RE-7-1 were found to be superior for total seed yield per plot and seed yield per plant. These trait donors can be further utilized for developing better genotypes through systematic hybridization.

**TABLE 4:** A path coefficient showing total, direct, and indirect effect of various key traits identified on seed yield per plant in mustard.

Traits	Indirect effect to SY/Plant									Total effects		Total correlation to SY/Plant (*r*)
			
	TSY/Plot	PH_FPB	BY/Plot	SMS	SS	TSC	DFI	PH_M	SL	Direct	Indirect	
**TSY/Plot (X_1_)**		0.05	0.02	0.07	0.09	0.01	–0.08	0.03	0.03	0.24	0.19	0.43
**PH_FPB (X_2_)**	–0.07		–0.01	–0.06	–0.07	–0.04	0.10	–0.10	–0.03	–0.19	–0.24	–0.42
**BY/Plot (X_3_)**	0.03	0.01		0.04	0.04	0.08	0.00	–0.03	0.01	0.16	0.20	0.36
**SMS (X_4_)**	0.07	0.05	0.03		0.03	0.05	–0.07	–0.01	0.01	0.23	0.15	0.37
**SS (X_5_)**	0.10	0.06	0.03	0.04		–0.03	–0.08	0.04	0.06	0.22	0.16	0.37
**TSC (X_6_)**	0.02	0.04	0.07	0.06	–0.03		–0.03	–0.02	–0.01	0.19	0.14	0.33
**DFI (X_7_)**	–0.11	–0.12	0.00	–0.09	–0.11	–0.03		–0.08	–0.04	0.16	–0.35	–0.19
**PH_M (X_8_)**	–0.05	–0.13	0.04	0.02	–0.05	0.02	0.08		–0.02	–0.15	–0.05	–0.20
**SL (X_9_)**	0.07	0.06	0.02	0.03	0.13	–0.02	–0.07	0.04		0.09	0.22	0.31

*SY/P, seed yield/plant; TSY/Plot, total seed yield/plot; PH_FPB, plant height up to first primary branch; BY/Plot, biological yield/plot; SS, seed size; TSC, total siliqua count; DFI, days to flowering initiation; PH_M, plant height at maturity; SL, siliqua length.*

**TABLE 5:** Promising genotypes and traits identified under study.

Favorable traits for breeding high yielding ideotype	Characters recorded	Acceptable limit*	Promising genotypes as donors with cluster information
Early flowering	DFI	<40 days	IC-766097 (Id), DRMRIJ 17-46 (Ie), DTM-25 (If)
Basal branching	PH_FB	Productive branches from <30 cm from ground	EM-1 (If), NRCHB-101 (If), IM-46 (1u), RNN-505 (1d), RH-819 (1f), EJ-22 (1d), IC-597894 (1f), NPJ-113 (1d), EJ-20 (1f), Pusa Mahak (1f), IC-261627 (In), IC-597949 (Im), NDR-8501 (Id), Shivani (Id), EC-61-3-1 (If), Sanjucta arech (If), RH-419 (If), DRMRIJ 17-38 (Ie), Pusa Mustard-28 (Id), DRMRIJ 17-46 (Ie), DRMRIJ 17-45 (If), NRCDR-2 (Id), IC-597867, NQM-5 (If), IC-597873 (Iv), Sitara Sagar (If), CN-105234 (If), Pusa Mustard -26 (Ie), IC-766097 (Ie), GM-1 (Ie), IM-24 (If)
Medium plant stature	PH_M	151–175 cm	DRMRIJ 17-42 (Id), IC-261627 (In)
No. of siliqua on main shoot	SMS	>60	IC-597885 (Im), JM-06010-1 (If), RNN-505 (Ie), Purbi Raya-1 (If), NPJ-190 (Id), RRN-443-2 (If), RLC-2 (If)
Bold seeds	SS	>6.0 g/1,000 seeds	RH-406 (It), RH-30 (Id), RH-725 (Id), RH-749 (Id), Ashavathi (Id), NAV-GOLD (Is)
Total siliqua count	TSC	>800/plant	CN-101846 (Iu), IC-597867 (IV), AJ-11 (Ia)
Siliquae length	SL	>5.25 cm	NRCDR-2 (Id)
Biological yield	BY/Plot	>30 t/ha	CS-54 (Id), Dingahini (Id), Pantnagar collection (Id), RGN-73 (Id), RNN-505 (Id)
Seed yield in population/community	TSY/Plot	>35 Q/ha	RGN-73 (Id), JM-3 (If), RH-725 (Id), RCQR-9901 (Ig), RE-13 (If), Shivalik (Id), PBR-210 (Id), RE-7-1 (Ic), RNN-505 (Id).
Seed yield per plant	SY/P	>30 g	IC-597867 (IV), PBR-210 (Id), RE-7-1 (Ic)

*Acceptable limit for each trait is fixed based on the various mustard ideotype concept given by Bhargava et al. (1984), VijayaKumar et al. (1996), DUS guidelines, Thurling (1991), and Yadav et al. (2017).

*Parenthesis indicates the subcluster details.*

### Diversity Analysis

**FIGURE 4:**
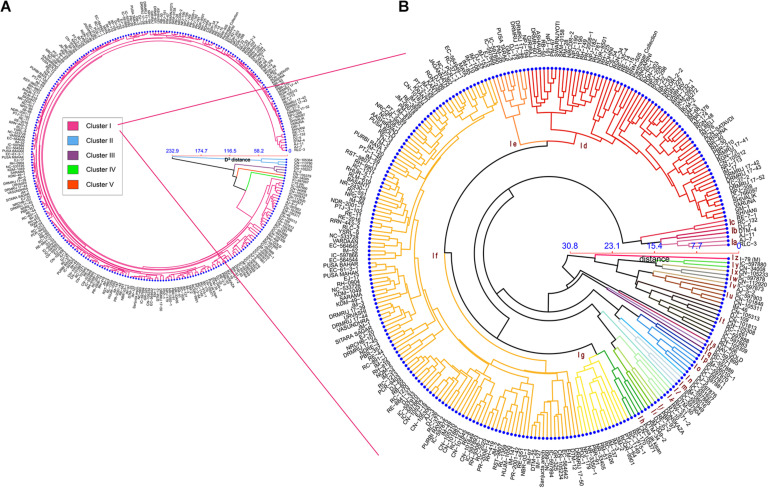
Dendrogram showing hierarchical clustering of 289 genotypes based on Mahalanobis’s distance and the UPGMA algorithm. **(A)** Circular dendrogram depicted 289 genotypes distributed in five clusters. **(B)** Detailed dendrogram of cluster I with 26 subclusters (coded as Ia–Iz).

Cluster analysis based on phenotypic data revealed that genotypes were distributed into five different clusters (I to V) at Mahalanobis *D*^2^ value of 60.0 (Figure 4A). *D*^2^ distance reported in the present study ranges from 0 to 351.38, also indicated huge genetic diversity in the population. Cluster I had 281 genotypes followed by clusters II, III (three genotypes each), IV, and V (one genotype each). Again, cluster I was found to have around 26 subclusters (a to z) at Mahalanobis *D*^2^ value of 20.0 (Figure 4 and Supplementary Table 2). Subcluster Ia contains three genotypes from the Australian gene pool; subcluster Ib contained two genotypes that were derivative of resynthesized *B. juncea.* Subcluster Ic consists of a breeding line from east European countries (2), and subcluster I (d–i) is dominated by high-yielding cultivars and advanced breeding lines of Indian origin. Most of the indigenous collection (IC) based on *B. juncea* var. *rugosa* and Canadian gene pool got together in subcluster I (j–z) (Figure 4B).

The relative contribution of each character indicated that seed size (13.14%), days to 50% flowering (10.85%), and siliqua length (7.88%) contributed maximum toward total divergence (Table 6). All the popular Indian cultivars got included in cluster I. Furthermore, it was explained that cluster I contained accessions producing the highest total seed yield per plot and seeds per siliquae, which are early and require a minimum for flowering traits. Genotypes belonging to cluster II were found to have a longer duration for flowering traits with higher harvest index and lowest values for several secondary branches, main shoot length, and total siliquae count. Cluster III is found to have genotypes having the higher plant height and biological yield per plot, whereas lower values for SL, SPS, and HI. Cluster IV contained a single genotype (IC-597867) having the highest seed yield per plant with higher MSL, SPS, and TSC with smaller seed size and shorter plant height up to the first primary branch. Similarly, cluster V contained a genotype (CN-34005) having maximum values for SS and SL and minimum values for PH_Fl, PH_M, PB, SY/P, TSY/Plot, and BY/Plot.

**TABLE 6:** Cluster means of 20 characters and its relative contribution evaluated for genetic divergence in 289 mustard genotypes in diversity panel.

Cluster	No. of genotypes	DFI	DFF	DHF	DCF	DMT	PH_FI	PH_FPB	PH_M	PB	SB	MSL	SMS	TSC	SL	SPS	SS	SY/P	TSY/Plot	BY/Plot	HI
I	281	52.02	58.16	67.19	101.86	141.49	120.18	49.47	223.42	6.26	13.01	77.40	50.78	484.90	3.55	14.41	3.56	17.94	661.55	5.07	15.13
II	3	123.41	131.99	136.71	151.40	171.58	120.52	66.28	215.77	6.46	12.70	34.07	37.58	360.61	2.93	13.86	2.26	13.58	567.62	4.71	15.22
III	3	107.30	116.52	125.51	140.17	170.34	211.08	148.89	269.37	7.17	13.09	34.84	33.49	449.65	2.41	12.87	2.33	9.04	443.44	5.25	11.86
IV	1	64.65	71.62	82.68	109.97	143.32	129.35	28.14	218.77	6.93	13.08	79.91	56.16	601.30	3.32	14.35	1.97	49.77	550.72	4.22	14.75
V	1	88.77	95.60	99.46	125.23	143.87	99.28	44.95	206.41	6.12	12.93	79.02	44.38	459.56	4.07	13.89	4.63	8.24	437.83	4.22	11.96
Singh statistic		163.75	257.78	56.40	155.30	175.73	118.82	174.40	92.27	82.35	45.56	83.04	73.07	71.00	187.35	49.34	312.24	109.56	67.32	59.13	42.53
% Contribution		6.89	10.85	2.37	6.53	7.39	5.00	7.34	3.88	3.46	1.92	3.49	3.07	2.99	7.88	2.08	13.14	4.61	2.83	2.49	1.79
Rank		6	2	17	7	4	8	5	10	12	19	11	13	14	3	18	1	9	15	16	20

*DFI, days to flowering initiation; DFF, days to 50% flowering; DHF, days to 100% flowering; DCF, days to flowering completion; DMT, days to maturity; PH_Fl, plant height at flowering (PH_Fl); PH_FPB, plant height up to first primary branch; PH_M, plant height at maturity; PB, number of primary branches; SB, number of secondary branches; MSL, main shoot length; SMS, siliquae on the main shoot; TSC, total siliquae count; SL, siliqua length; SPS, seeds per siliqua; SS, seed size; SY/Plant, seed yield/plant; TSY/Plot, total seed yield/plot; BY/Plot, biological yield/plot; HI, harvest index.*

Although genotypes in cluster I were from the different geographical locations, genotypes belonging to the same location or same breeding program that tend to fell together in the same subclusters such as DRMRIJ-17 series, i.e., 17-41,17-42, 17-43, and 17-52 derived from ICAR-Directorate of Rapeseed and Mustard Research, Bharatpur, fell on subcluster Id of cluster I. Similarly, the indigenous collections from Arunachal Pradesh, such as IC-597870, IC-597871, and IC-597881 (subcluster Io) and IC-597904 and IC-597949 (subcluster In), and most of the Canadian gene pool from subclusters I (t–y) followed the same pattern as the DRMRIJ series. In contradictions to the observation discussed earlier, a few accessions collected from the same region, such as Canadian germplasms, did not fell in a single cluster or subcluster, indicating that geographical proximity does not always result in genetic similarity.

**FIGURE 5:**
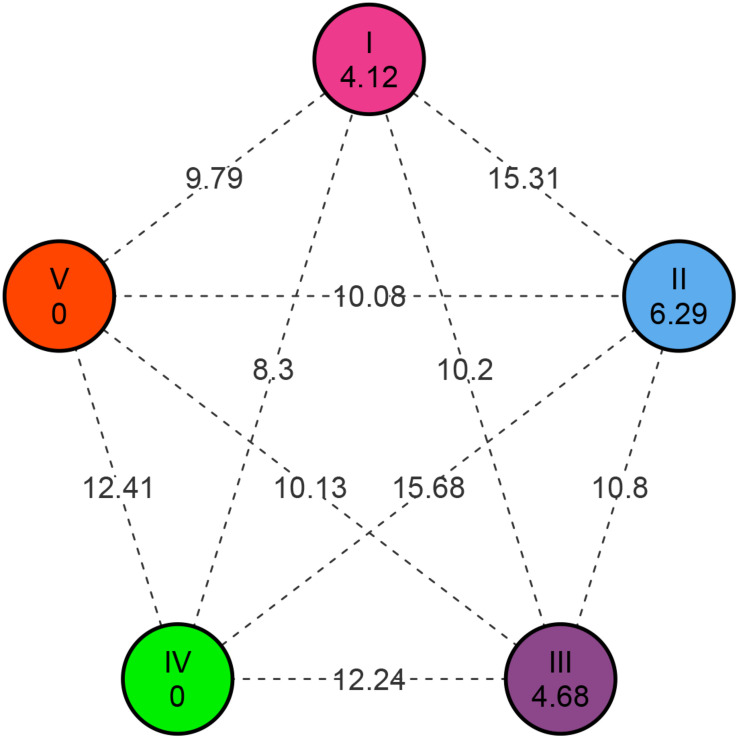
Intra- and inter-cluster distance based on *D*-value for Indian mustard genotypes.

Based on the similarity matrix, the distance between clusters, inter-cluster *D*-values ranged from 8.30 to 15.68 (Figure 5). The higher inter-cluster distances than the intra-cluster distances designated wider genetic diversity of different groups among the genotypes. Clusters II and IV were strikingly diverse from the rest of the clusters (inter-cluster *D*-value = 15.68); therefore, intercrossing the genotypes from these two clusters may create wider variability and is estimated to throw high yielding transgressive segregants in the mustard breeding program. The minimum inter-cluster *D*-value (8.30) detected between clusters I and IV showed the higher genetic similarities between these clusters. Intra-cluster distance (*D*) revealed that cluster II showed maximum intra-cluster distance (6.29) followed by cluster III (4.68). Owing to solitary genotype, clusters IV and V did not show intra-cluster distance. The magnitudes of the intra-cluster distances were not always proportional to the number of genotypes in the cluster, as intra-cluster distance in cluster I was found to be moderate (4.12) regardless of maximum genotypes (281).

## Discussion

The exploitation of genetic diversity present in a species can lead to the improvement of traits of the economic importance of mandated crops with the intervention of plant breeders to benefit the farmers and consumers (Salgotra et al., 2015). Yield is one of the most important economic traits and is the product of multiplicative interactions of contributing characters (Kant and Gulati, 2001). To combat these complex interactions, we need to have a multipronged strategy by combining agronomical and breeding approaches. Hence, the major objective of the mustard improvement program is to develop varieties with high yield potential through the introgression of various yield component traits from the lines with high trait values. Hence, exploitation of germplasm lines to identify lines with higher trait values is of prime importance.

*Brassica juncea* is a major oilseed crop of *Rabi* (winter) season and is highly sensitive to weather parameters such as temperature and solar radiation, which affect growth, phenological events, and crop yield (Kumar, 2005). Intermittent rains during the flowering time of 2018–2019 caused substantial yield losses by physiological disorder and biotic stresses. Therefore, a large variation in yields from year to year can be attributed to the weather conditions. Mustard prefers moderate temperatures between 18 and 25°C with an optimum around 20°C and moderate rain of approximately 25–40 cm during the growth period (Bhatt et al., 2015). The sensitive periods for mustard crop growth signify emergence, flowering, siliqua formation, siliqua filling, and physiological maturity. The analysis revealed that maximum and minimum temperatures had a positive effect on the yield during the sensitive period in both seasons, whereas total rainfall had a negative effect on the mustard yield during 2018–2019. The intermittent rainfall resulted in high RH (>92%) with *T*_*max*_ ranging from 18.7 to 24.4°C recorded from 4 to 10 standard meteorological weeks during the reproductive period of the plant (88–128 DOS) resulted in subsequent yield loss.

### Mean Performance and Variation for Phenotypic Traits

Seed yield and related traits showed wide phenotypic variations during both seasons. The mean performance indicated the existence of enormous variability for the seed yield and related components, which offer greater opportunities for utilizing these traits in future breeding programs (Kumar et al., 2020). The genetic variation available for traits such as total seed yield per plot, total siliquae count, plant height at first branching, etc., can meet the breeding objective in evolving a high-yielding *B. juncea* cultivar. The greater variability observed in the present study could be due to the use of genotypes from diverse geographical origins. The lowest coefficient of variation for the number of days to maturity (1.80%) showed its best genetic potential and genetic influence, whereas the highest coefficient of variation for harvest index (30.0%) showed more influence of environmental fluctuations (Khan et al., 2008). Some of the yield traits such as total siliqua count, seed yield per plant, total seed yield per plot, biological yield, and harvest index showed a greater CV above 20%, which may be due to the longer duration and photoperiod sensitivity of some exotic lines, especially the Canadian lines. Characters with extensive genetic variability provide a better opportunity for selection instead of those with a narrow range of variability. Ali et al. (2003); Yadava et al. (2011), and Roy et al. (2018) had also found significant genetic variation as indicated by range for different seed yield-contributing characters in Indian mustard but comparatively lower than the present study.

The analysis of variance revealed highly significant differences for all the characters representing the presence of variability, which can be utilized through genetic improvements. Significant variance due to G × E interactions for all the 20 characters confirmed that the genotypes respond differently in diverse environments. Therefore, it is possible to exploit different environments by developing environmentally specific varieties from the diversity panel. The importance of G × E interactions had also been observed by Gunasekara et al. (2006); Kumar et al. (2012), Priyamedha and Haider (2017), and Kumari et al. (2019) in Indian mustard and canola (*B. napus*) for seed yield.

### Significance of Heritability and Other Genetic Parameters for the Selection of Traits

The observed variation in a population may be either due to genetic or environmental or both. Only those due to genetic components remain heritable. Heritability alone does not infer the estimate of the actual amount of genetic gain in the selection program, as it is also inclusive of nonadditive genetic factors (Shivanna, 2008). All the yield traits in the current study were highly heritable in individual seasons. Nevertheless, a partitioned genotype by environment interaction decreased the heritability across environments (pooled analysis). This type of reduced heritability across the environment was also reported by Phuke et al. (2017) in Sorghum.

The study showed that high broad-sense heritability, genetic advance as percentage mean, and GCV were observed in flowering traits such as days to flowering initiation, days to 50% flowering, days to 100% flowering, plant height at flowering initiation, plant height up to the first primary branch, seed size, and seed yield/plant. The very high heritability of seed yield per plant (63%) in pooled analysis with a high GA of 60.8% indicated that the results would be repeatable and rewarding over generations of selection cycles, which is a boost for the breeding program. This concurred with previous studies (Kumar and Misra, 2007; Yadava et al., 2011; Tripathi et al., 2013; Meena et al., 2017; Kumar et al., 2019). These results indicate a greater scope for selection to improve seed yield *per se* in the *Brassica* breeding program (Tiwari et al., 2017).

Estimates of heritability for yield component traits varied from low (22%) to high (85%). There is a need to identify the traits that should be targeted for improving the seed yield in mustard. Flowering traits, plant height traits, and seed size showed high heritability and high GA, as few of them are governed by a few major quantitative trait loci reported earlier by Kaur and Banga (2015) and Akhatar et al. (2021). The high value of heritability and moderate genetic advance for plant height at maturity indicated that improvement in this trait could be made through the selection to some extent. High genetic advance and moderate heritability were shown in the number of primary branches, total siliquae count, total seed yield/plot, and biological yield/plot in which both additive and nonadditive gene actions may be expressed. A parameter having low heritability coupled with high genetic advance revealed the additive gene effects of traits (Belete et al., 2011). The low heritability is due to high environmental effects, and selection *per se* may be ineffective for such traits as harvest index in the present study. None of the traits exhibited low heritability with low genetic advance.

Higher PCV values than their corresponding GCV for most of the traits showed the considerable role of environment in the expression of these traits; therefore, the variation in the genotypes is due to both genotype and the environment (Kumar et al., 2015). The wide difference between PCV and GCV was detected for plant height up to the first primary branch, the number of secondary branches, total siliquae count, seed yield/plant, total seed yield/plot, biological yield/plot, and harvest index, which indicated the high contribution of environmental variance to the phenotypic variance.

### Association Among Traits

Correlation analysis indicated that seed yield per plant was significantly correlated with biological yield/plot, total seed yield/plot, seed size, plant height up to the first branch, total siliqua count, days to flowering initiation, plant height at maturity, siliqua length, and siliqua on the main shoot, which implies that selection in improving these traits would lead to improved seed yield (Rauf and Rahim, 2018). Genotypic correlations involving flowering characters such as days to flowering initiation, days to 50% flowering, days to 100% flowering, days to flowering completion, days to maturity, and plant height characters such as plant height at flowering initiation, plant height up to the first primary branch, and plant height at maturity with seed yield for plants in studied genotypes were negative, indicating selection for these traits would decrease seed yield (Joshi et al., 2009; Yadava et al., 2011). Reduction in flowering days prevents plant exposure toward high temperature that builds up during the late growth periods and consequent reduction in yield due to sterility and shriveling of seeds (Azharudheen et al., 2013). Also, reduced plant height makes plant architecture more compact to utilize the source toward increment in yield. These attributes can serve as marker characters for seed yield improvement in mustard. According to Kardam and Singh (2005), characters such as height up to the first branch, seed yield/plant, number of primary branches, number of siliquae per plant, and seed size have been reported as main yield contributing traits.

For instance, the number of secondary branches with very low heritability was significantly positively correlated to high heritability traits *viz.* total siliquae count (*r* = 0.57) and biological yield/plot (*r* = 0.33). Therefore, the selection of genotypes with higher siliqua count and biological yield/plot would indirectly improve the number of secondary branches per plant in successive generations. This is in accordance with the findings of Prasad et al. (2001), Swarnkar et al. (2002), and Singh et al. (2002).

Regression is a method for automatic selection in a stepwise manner based on partial correlations of a dependent variable with the independent variables near to optimal in the sense of maximizing the squared multiple correlations coefficient (*R*^2^) of the dependent variable (Card et al., 1988). Based on regression studies, biological yield/plot, total seed yield/plot, plant height up to the first primary branch, seed size, total siliquae count, number of days to flowering initiation, plant height at maturity, siliquae on the main shoot, and siliqua length were the most contributing traits for seed yield per plant. However, these independent traits with individual *R*^2^ of less than 20% had only a negligible direct contribution to the seed yield per plant, although they had a significant association with the dependent variable. This indicates that those traits that had less direct influence cannot be ignored because their cumulative contribution to seed yield/plant could be highly influential (Maphumulo et al., 2015). These identified traits in combination could be used as effective indicators in Indian mustard for the calculation of yield performance; hence, a selection index based on identified influential traits could realize higher genetic advances than selecting seed yield alone (Hussain et al., 2004; Sandhu et al., 2019).

### Contribution Toward Seed Yield per Plant

Specific direct and indirect effects of traits and relative importance of each trait in determining the key goal, i.e., seed yield, was accompanied through path coefficient analysis (Albayrak and Tongel, 2006). Path analysis that showed total seed yield/plot, siliquae on the main shoot, and seed size had a highly positive correlation and moderate direct effect on seed yield per plot, which suggested that selection for these traits would be quite effective for improving seed yield in mustard. Traits such as plant height up to the first primary branch and plant height at maturity had a negative moderate direct effect. Similar conclusions were reported by Kardam and Singh (2005) and Sandhu et al. (2019). Indirect effects were ranked similar to those of Lenka and Mishra (1973), as follows: 0.00–0.09 = negligible, 0.10–0.19 = low, 0.20–0.29 = moderate, and >0.30 = high path coefficients. Plant height at maturity toward dwarf plant type exhibited negligible indirect effects on seed yield, indicating that they had little contribution to seed yield. The rest of the traits were low to moderate, showing that indirect selection for these traits would improve the yield of the mustard. Hence, for improving the seed yield per plant in mustard, one should focus on “selecting for” traits such as total seed yield per plot (under crop community), more siliquae on the main shoot, bold seed size, and highest total siliquae count per plant and “selecting against” plant height up to the first primary branches (i.e., selection for basal branching genotypes). Traits such as days to flowering initiation, plant height up to the primary branches, siliquae length, and biological yield have significant indirect effects *via* component traits toward seed yield per plant. The traits as mentioned earlier with high direct effects inferred from path analysis such as total seed yield/plot, seed size, siliqua on the main shoot, plant height up to the first primary branch, and plant height at maturity had moderate to high heritability (≥30%) coupled with high GA% of the mean (>20%). These traits also showed a highly significant (*p* ≤ 0.001) correlation with seed yield/plant. Table 5 summarizes promising genotypes identified based on the cumulative performance for yield and associated traits that can be a guide in bringing high-yielding ideotypes in mustard for the entire mustard breeding community.

### Diversity Analysis

The advantage of genetic diversity analysis based on Mahalanobis *D*^2^ distance over the Euclidian distance is that it can take account of the correlation between a highly correlated variable and can scale the contribution of individual variables to the distance value according to the variability of each variable (Ghorbani, 2019). The Mahalanobis *D*^2^ distance among genotypes in the diversity panel ranged from 0.0 to 351.38, which was huge and higher than previous reports by Bind et al. (2015); Gupta et al. (2015), and Chandra et al. (2018). The huge extent of genetic diversity in the present study was due to the involvement of genetic material from four continents across the globe. Furthermore, the number of genotypes studied was higher compared with the previous reports. Most of the genotypes (281/289) got included in a single cluster and the rest of them in four different clusters. The cluster forming point was having a very high *D*^2^-value = 232. It suggests that the eight genotypes included in clusters II to V were more diverse than cluster I. Also, a detailed analysis of cluster I also suggested that there were 26 subclusters with a cluster-forming point at a *D*^2^ value of 20. Intra-cluster distance of cluster I (*D*^2^ = 17) was much higher than earlier reports related to *D*^2^ clustering studies by Yadava et al. (2011) and Kumari and Kumari (2018), indicating a wider genetic base of materials within cluster I in the present study. The subcluster I (d–i) included most of the high-yielding cultivars and breeding lines of Indian origin. The rest of the subclusters consisted of exotic and indigenous gene pools received in various bilateral collaborative projects utilized in mustard improvement by various researchers (Chauhan et al., 2011). Subcluster I (j–z) consisted of lines having more height and longer duration with various oil quality traits, which remain unexploited due to lack of synchrony in flowering time. Still, there was no obvious clustering pattern related to geographic proximity and use types among mustard. Grouping of certain improved varieties and cultivars along with Canadian and Australian genetic stocks and indigenous collections from Arunachal Pradesh (India) indicated that the geographical distribution need not necessarily be the indicator of genetic divergence as reported by Verma and Sachan (2000) and Jeena and Sheikh (2003). The possible reason could be the common ancestry of these genotypes, which permitted the free exchange of germplasm among the breeders of different locations and/or the unidirectional selection experienced by breeders in tailoring the promising cultivars for different locations (Yadava et al., 2011; Mukesh Sankar et al., 2014).

Broad variability in the current material holds great promise to use these genotypes from different clusters (such as II and IV) for future breeding programs. The highest-yielding genotype (IC-597867) identified in the present study was present in cluster IV, whereas genotypes with a high harvest index were contained in cluster II. So, the improved cultivars from cluster I can be utilized to exploit the genotypes present in clusters II and IV for further yield increments and genetic diversification through hybridization.

## Conclusion

The study assessed a diversity panel representing 289 genotypes across four continents for the existence of genetic variability for seed yield and yield-related traits over two seasons. The results revealed an enormous genetic variability for the traits under study, which can be exploited to acquire further breeding gains. The use of BLUP values for genotypes provided higher selection accuracy by reducing residual error, which permitted the identification of potential genotypes for the *Brassica* improvement program. Flowering and plant height-related traits were found to be more heritable, although these were negatively correlated with yield. High PCV, GCV, and genetic advance values with low to moderate heritability were observed for total seed yield, which indicated that the yield performance still needs to be improved to produce superior varieties. Moreover, the current study could aid breeders in enhancing the seed yield by considering the traits that have a good correlation with seed yield. Cluster analysis revealed that genotypes under study were more diverse, which could be utilized for future hybridization programs, and it can release transgressive segregants for economic trait improvement. The greater variability among the studied genotypes and the association between seed yield and secondary traits in the current study suggested the exploitation of superior genotypes in the near future.

## Data Availability Statement

The original contributions presented in the study are included in the article/Supplementary Material, further inquiries can be directed to the corresponding author.

## Author Contributions

DY conceptualized, supervised the research, and contributed to reviewing the manuscript. RS and SLS performed the field trials and prepared the original draft manuscript. SS performed first-year field trial. SM conducted the data analysis, curated the data, and contributed in reviewing the manuscript. RC helped in field trials and data entry. YP and NS coordinated the study and revised the manuscript. SV administered the project and revised the manuscript. All authors read the manuscript and agreed with its content.

## Conflict of Interest

The authors declare that the research was conducted in the absence of any commercial or financial relationships that could be construed as a potential conflict of interest.
